# Thermoregulation and Heat Stroke Prevention in Older Adults: Advances in Emerging Technologies and Interventions

**DOI:** 10.3390/s25227058

**Published:** 2025-11-19

**Authors:** Sandra Núñez-Rodríguez, Carla Collazo-Riobó, Félix Menéndez-Vega, Javier Sedano, Ana Isabel Sánchez-Iglesias, Jerónimo Javier González-Bernal, Josefa González-Santos

**Affiliations:** 1 Faculty of Health Sciences, University Isabel I, 09003 Burgos, Spain; sandra.nunez@ui1.es; 2 Department of Health Sciences, University of Burgos, 09001 Burgos, Spain; fmv1002@alu.ubu.es (F.M.-V.); asiglesias@ubu.es (A.I.S.-I.); jejavier@ubu.es (J.J.G.-B.); mjgonzalez@ubu.es (J.G.-S.); 3 Instituto Tecnológico de Castilla y León, 09001 Burgos, Spain; javier.sedano@itcl.es

**Keywords:** older adults, thermoregulation, wearable devices, cooling strategies, thermal comfort, heat stress prevention, systematic review

## Abstract

This systematic review explores interventions and technologies aimed at assessing or improving thermoregulation in older adults (≥60 years) across community, clinical, and institutional settings. Following PRISMA guidelines, a comprehensive search in PubMed, Scopus, Web of Science, and ScienceDirect (2010–2025) identified 449 records, of which nine studies met inclusion criteria. The evidence revealed that thermoregulation in older adults is influenced by complex interactions between environmental, physiological, behavioral, and cognitive factors. Predictive models based on skin or facial temperature, and supported by machine learning approaches, showed promising accuracy in estimating thermal sensation and core temperature. Active interventions, such as cooling devices, ventilated clothing, and microclimatic systems, demonstrated significant reductions in skin and core temperature, improving thermal comfort. Educational and community-based strategies also proved effective in reducing thermal risk perception and adverse events. However, despite the availability of wearable devices capable of continuous monitoring, no studies have reported their application in daily-life or environmental contexts for heatstroke prevention in older adults. This gap highlights a critical opportunity for integrating wearable technologies into preventive frameworks. Future research should focus on validating these approaches in ecological settings and tailoring strategies to individual vulnerabilities, thereby improving thermal safety, autonomy, and well-being in this vulnerable population.

## 1. Introduction

**Figure 1:**
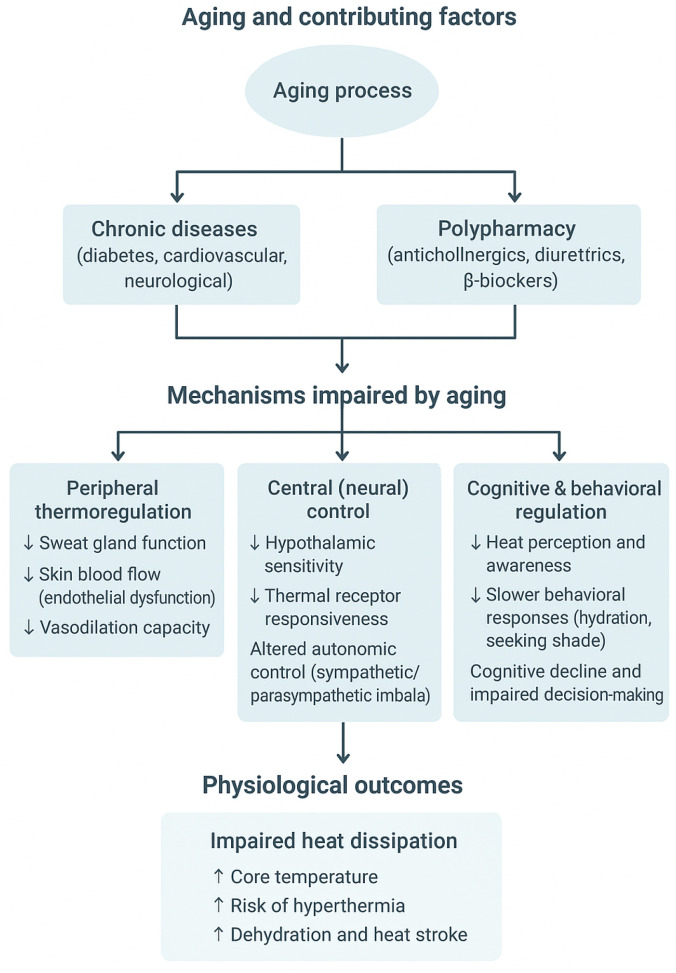
Mechanisms linking aging and impaired heat dissipation.

Thermoregulation, defined as the set of physiological processes that maintain a stable body temperature, is fundamental for the health and well-being of older adults [1]. With aging, these mechanisms undergo significant changes, including reduced sweating, decreased peripheral vasodilation, diminished perception of thermal stress, and alterations in cardiovascular and autonomic function [2,3]. These physiological modifications, combined with common comorbidities, polypharmacy, and exposure to extreme environments, increase older adults’ vulnerability to adverse thermal events such as hyperthermia, hypothermia, hospitalization, and even mortality. Exposure to extreme heat events represents a significant public health concern, with a growing impact due to climate change and the increasing proportion of the population with reduced capacity to maintain thermal homeostasis [4]. At the physiological level, aging affects both central and peripheral thermoregulatory mechanisms (Figure 1). The hypothalamic set-point for heat dissipation becomes less responsive, while peripheral thermoreceptors show reduced sensitivity, delaying the onset of vasodilation and sweating [2,5,6]. Endothelial dysfunction limits cutaneous blood flow, and reduced cardiac output further restricts heat transfer from the core to the skin [7,8]. Muscular atrophy and diminished metabolic rate also decrease heat generation in cold environments [1,9]. In addition, cognitive and perceptual changes associated with aging—such as impaired thermal awareness, slower decision-making, and medication-related effects on autonomic function—compromise behavioral thermoregulation. Together, these processes explain the reduced ability of older adults to maintain internal temperature stability under thermal stress [1,5].

Although many heatstroke cases occur among essential workers exposed to high temperatures, the highest mortality rates are consistently observed among older adults [10,11,12]. This group is particularly vulnerable due to impaired thermoregulatory mechanisms, diminished thermal awareness, chronic diseases, and medication use that hinder physiological adaptation. Therefore, continuous monitoring technologies represent a promising strategy for daily prevention and early detection of heat-related risks in this population [13].

Thermal risk in older adults is not limited to clinical settings; it is also observed in homes and community environments, where factors such as housing insulation, access to air conditioning, availability of drinking water, and social support influence both exposure and coping capacity [14,15]. This complexity implies that the prevention of adverse thermal events requires an integrated approach that considers not only environmental conditions but also individual and behavioral factors. Some evidence suggests that older adults may occasionally underestimate their thermal sensation or show delayed behavioral responses to mitigate risk, highlighting the need for objective and continuous monitoring strategies [16,17].

In response to this vulnerability, various strategies and technologies have been developed to assess and improve thermoregulation in older adults. These include wearable devices capable of continuously monitoring body temperature and other physiological indicators, smart clothing with thermoregulatory properties, community interventions during heat or cold waves, and clinical protocols for cooling, hydration, or environmental conditioning. Among wearable technologies specifically designed for thermoregulation, three main categories can be distinguished: (i) thermoelectric devices (TEDs), which use the Peltier effect to locally cool or heat the skin, achieving temperature reductions of up to 10 °C while enabling personalized control with high energy efficiency [18,19,20]; (ii) smart textiles and garments, which incorporate advanced fibers and materials capable of reflecting, dissipating, or retaining heat, with some models integrating embedded sensors for automatic monitoring and regulation [21,22,23,24,25]; and (iii) sweat patches and biosensors, which allow continuous monitoring of perspiration and related physiological markers, thereby facilitating early detection of thermal imbalances [26,27,28,29]. A distinction should be made between smart clothing, which refers to garments that incorporate sensors or actuators to dynamically control or regulate temperature, and smart fabrics, which typically describe fabrics designed with intrinsic thermoregulatory properties (e.g., phase-change materials or reflective fibers) without integrated electronic components. These interventions and devices aim to detect early changes in body temperature, prevent adverse events, and promote the thermal well-being and safety of older adults in community, clinical, and institutional settings.

The impact of these interventions extends beyond physiological outcomes. Adequate monitoring and the implementation of preventive strategies can reduce hospitalizations, complications, and mortality associated with thermal stress, while also supporting autonomy, social participation, and overall well-being in older adults. Emerging technologies, such as wearables, enable continuous and personalized data collection, offering potential for the development of early warning systems and preventive strategies tailored to individual characteristics [30,31,32,33].

Furthermore, the diversity of approaches and technologies provides an opportunity to explore which tools are most effective and adaptable to different contexts, from clinical care to community environments, taking into account environmental, physiological, behavioral, and social factors. Accumulated evidence suggests that combining precise monitoring, educational interventions, active cooling strategies, and community programs can enhance thermal safety in older adults, although the relative effectiveness of each approach may vary depending on context and population characteristics [34].

Therefore, this systematic review aims to identify, describe, and critically evaluate interventions and technologies designed to assess or improve thermoregulation in adults aged 60 years and older. The review focuses on their application in community and institutional settings, analyzing their impact on temperature monitoring, prevention of heat-related adverse events, and promotion of thermal well-being in daily life. By synthesizing current evidence, this study seeks to inform future technological adaptations and preventive strategies tailored to the specific vulnerabilities of the aging population.

## 2. Materials and Methods

This systematic review was carried out following the PRISMA Statement guidelines and adhering to a predefined research protocol. The comprehensive literature search was performed between 26 July 2025 and 4 August 2025, consulting the electronic versions of PubMed, Scopus, Web of Science, and ScienceDirect. Initially, the research question was formulated in PIO format, in accordance with the criteria proposed by Sackett et al. (1996) [34]. Table 1 shows the corresponding PIO format. The PRISMA checklist can be found in the Supplementary Information (Table S1). 

Subsequently, using this format and the PIO question as a framework, tailored search strategies were developed and adapted for each of the databases consulted. The search strategies are shown in Table 2.

Original research studies were included if they met the following criteria: (1) employed an experimental (controlled trials, laboratory studies) or observational design (cohort, case–control, time series, ecological studies, or case reports); (2) were published from 2010 onwards; (3) had at least the abstract available; and (4) evaluated interventions or technologies aimed at assessing or improving thermoregulation (such as wearable sensors, smart clothing, cooling or hydration protocols, or community-based programs during heat or cold events) and/or reported outcomes related to body temperature monitoring, early detection of thermal alterations, prevention of heat- or cold-related adverse events, or enhancement of thermal safety and well-being in older adults (≥60 years) in community, institutional, or clinical settings.

Exclusion criteria encompassed narrative reviews, systematic reviews, meta-analyses, letters to the editor, or low-quality publications, as well as studies that did not address the research question, did not include adults aged 60 or older, or exclusively examined animals, in vitro models, or pharmacological interventions unrelated to thermoregulatory outcomes.

Additionally, a manual backward search, or snowballing, was conducted to capture potentially relevant studies not identified in the initial database search. Reference lists of included studies and sources of gray literature were also reviewed.

Study selection was performed in two sequential steps using Rayyan 1.4.3 software [35]. In the first step, titles and abstracts were screened to remove clearly irrelevant records. In the second step, the full texts of potentially eligible studies were evaluated. Both stages were carried out independently by two reviewers, with a third reviewer consulted to resolve any discrepancies or uncertainties.

To maintain consistency in data extraction, a standardized data collection form was developed, capturing details such as authors, year of publication, country, study design, sample size and participant characteristics, interventions or technologies applied, outcomes measured, instruments or tools used, key findings, and main conclusions. The thermophysiological variables reported in the included studies were measured following standardized recommendations for the evaluation of thermal strain (ISO 9886:2004) [36]. Core and skin temperatures were commonly obtained with rectal, tympanic, or surface thermometers validated for human studies, such as the Braun ThermoScan LF 40 (Braun GmbH, Jronberg im Taunus, Germany) [37]. Thermal indices frequently applied across studies, including the Predicted Mean Vote (PMV) [38], the Adaptive Comfort Model [39], and the Universal Berkeley Comfort (UCB) model [40], were cited according to their original formulations. Indices for occupational heat risk such as the Heat Strain Score Index (HSSI) [41] and the Observational–Perceptual Heat Strain Risk Assessment (OPHSRA) [42] were also referenced to their source publications.

The methodological quality and risk of bias of the included studies were assessed using the Joanna Briggs Institute critical appraisal tools, applying the version appropriate for each study design (controlled trials, cohort, case–control, time series, ecological studies, or case reports). A minimum quality threshold of 7 points was required for inclusion, and a pilot assessment was conducted among reviewers to ensure uniform application of the criteria.

Due to the marked heterogeneity in study designs, interventions, and outcome measures, a quantitative meta-analysis or heterogeneity assessment was not feasible. Instead, a narrative synthesis was conducted, supported by a descriptive comparison of the main quantitative indicators (e.g., reduction in skin/core temperature achieved by cooling interventions, and prediction accuracy or RMSE values in machine learning models).

## 3. Results

**Figure 2:**
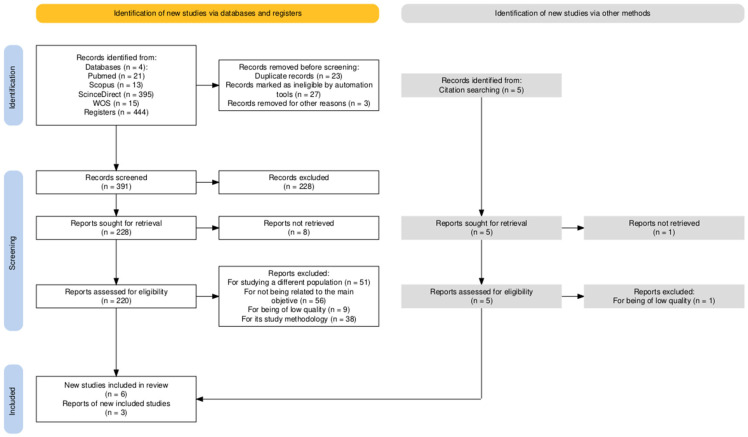
Flow-diagram for study selection.

A total of 449 results were identified in the initial search. After critical evaluation of the full text, 9 articles were selected for this review. Figure 2 shows the PRISMA flow diagram outlining the study selection process.

Table 3 also summarizes the main characteristics and key findings of the included studies, along with their methodological quality scores assessed using the Joanna Briggs Institute (JBI) critical appraisal tool.

### 3.1. Study Characteristics

A total of nine studies were included in this systematic review, comprising eight non-randomized experimental studies [43,44,45,46,47,48,50,51,52] and one observational study [49]. The experimental research primarily evaluated the applicability of physiological and environmental monitoring tools, predictive modeling, and innovative cooling or comfort systems in older adults, whereas the observational study investigated behavioral and adaptive strategies for heat prevention in community-dwelling populations [49].

The studies were geographically distributed across Asia (n = 4, 44.4%) [43,47,49,50], Europe (n = 2, 22.2%) [44,51], North America (n = 2, 22.2%) [45,52], and the Middle East (n = 1, 11.1%) [48]. Sample sizes varied substantially, ranging from small experimental groups of 8–34 participants in laboratory settings [47,52] to large-scale community-based cohorts exceeding 1000 older adults [43]. The age of participants ranged from 60 to 101 years, with several studies focusing on institutionalized populations, including nursing home residents [44,48] or individuals with dementia [52], while others recruited community-dwelling older adults [43,49,50]. Sex distribution was reported in most studies, though balance between men and women varied.

Physiological and environmental assessments included core temperature measured via rectal or tympanic thermometry [47,50,52], skin temperature using thermistors or infrared thermography [44,51,52], and wearable sensors enabling continuous monitoring [45,48]. Environmental variables such as ambient temperature, relative humidity, air velocity, and radiant heat were consistently monitored across experimental protocols [43,44,50]. In addition, several studies integrated computational approaches, including predictive algorithms and machine learning models, to estimate thermal comfort or core temperature without invasive measurement tools [44,45,50].

Interventions investigated in the included studies encompassed personal cooling strategies such as fans, ventilated clothing, or microclimate chairs [47,53], predictive models to optimize monitoring and early detection of heat stress [44,45,50], and institutional or community-based adaptive strategies during heat waves [48,49]. These studies were conducted in both controlled laboratory environments and real-world settings, reflecting diverse approaches to assessing and improving thermoregulation in older adults.

Overall, the included studies showed considerable heterogeneity in design, population, and methodology. Despite these differences, all shared the common aim of exploring strategies, technologies, or programs to support thermoregulation in older adults across community, institutional, and clinical contexts.

The methodological quality and risk of bias of the included studies were assessed, with all of them obtaining scores above the established cut-off for their design (Table 4 and Table 5).

### 3.2. Description of the Characteristics of the Studies

The aim of this systematic review was to examine interventions and technologies implemented in older adults (≥60 years), including those living in community, institutional, or clinical settings, to assess or improve thermoregulation. The focus was on strategies such as wearables, smart clothing, community-based programs during heat waves, and cooling or hydration protocols. The review sought to evaluate their impact on body temperature monitoring, early detection of thermal alterations, prevention of heat- or cold-related adverse events, and overall promotion of thermal well-being.

The integrated analysis of the reviewed studies indicates that thermal regulation and sensation in older adults are influenced by a complex interplay of environmental, physiological, behavioral, and cognitive factors. The evidence demonstrates that the prediction of heat or cold perception depends on both environmental conditions and individual physiological responses, emphasizing the need for personalized approaches to thermal monitoring in this population.

Although no formal meta-analysis was performed, a comparative summary of the quantitative effects across studies was undertaken. Cooling interventions (fans, ventilated clothing, and microclimate chairs) reduced local skin temperature by 0.5–3.4 °C and core temperature by up to 1.7 °C, improving thermal sensation from ‘slightly warm’ to ‘neutral.’ Predictive models based on machine learning achieved mean RMSE values of 0.27 °C for rectal temperature and 0.73 °C for skin temperature, outperforming classical biophysical models.

Predictive models of thermal sensation highlight the primary role of air temperature, with additional contributions from factors such as air velocity, illuminance, and CO_2_ concentration [43]. Multi-site skin temperature measurements, particularly at the head and forearm, enhanced predictive accuracy, achieving 76.7% in laboratory settings and outperforming traditional PMV and UCB models. Complementary findings identify facial temperature as a robust indicator of thermal perception, with the nose emerging as the most informative site, followed by the cheek, forehead, and chin, and significant regional differences observed [47].

Machine learning approaches have further advanced individualized prediction. Ridge and linear regression models demonstrated low RMSE values (0.27 °C for rectal temperature, 0.73 °C for skin temperature), while sequential models such as GRU achieved the highest accuracy in dynamic scenarios [45,52]. Individual characteristics, including age, height, and weight, were shown to influence predicted body temperature, whereas elevated ambient temperature and humidity increased predicted thermal load, highlighting the interaction between personal and environmental factors.

Behavioral evidence suggests variability in exposure regulation. Residents with dementia tended to adjust clothing less and occupy slightly cooler spaces, whereas non-dementia residents modified attire in response to ambient conditions, maintaining relative comfort [44]. During extreme heat events, indoor temperatures in older adults’ homes reached nearly 30 °C, and although air conditioner use increased substantially, a significant portion of residents did not utilize cooling, indicating sustained exposure and limitations in passive strategies [49]. Educational interventions addressing hydration, breaks, protective clothing, and recognition of heat stress effectively reduced risk, increasing the “safe” category in HSSI and reducing “very high” risk in OPHSRA [48].

Active cooling strategies also proved effective. Local cooling devices and microclimate systems with ventilated vests substantially reduced skin temperature and perceived thermal load, especially in exposed regions such as the torso, arms, and hands, with internal temperature reductions of up to 3.4 °C [50,51]. Computational models closely matched experimental measures (±0.5 °C temperature, ±0.2 m/s air velocity), validating their application for assessing thermal mitigation strategies.

Overall, the evidence reveals that thermal perception and regulation in older adults are highly individualized and multifactorial. Integrating predictive modeling, targeted skin measurements (head, forearm, nose, cheek), and active cooling strategies provides a promising framework for mitigating heat exposure risks. Importantly, no studies were found applying wearables directly to prevent heatstroke in real-world environments, despite the strong potential for these technologies to support personalized preventive interventions combining physiological, environmental, and behavioral data to protect this vulnerable population.

## 4. Discussion

This systematic review aimed to analyze the evidence on interventions and technologies designed to assess or improve thermoregulation in older adults, focusing on monitoring, prevention of adverse thermal events, and promotion of thermal well-being. The integrated findings from nine studies reveal a multifactorial and highly individualized profile of thermal perception and temperature regulation in this population, influenced by environmental, physiological, behavioral, and cognitive factors. This complexity implies that any prevention strategy must consider the interaction between these factors and adapt to the individual characteristics of each older adult.

Thermal sensation predictive models consistently demonstrated that ambient temperature is the primary determinant of thermal perception, with additional contributions from air velocity, lighting, CO_2_ concentration, and individual physiological indicators such as localized skin or facial temperatures [43,47]. Measuring skin temperature at multiple sites, especially the head and forearm, significantly improved model accuracy, surpassing traditional approaches like PMV (Predicted Mean Vote) and UCB (Universal Climate Index). Facial temperature, particularly at the nose, emerged as a robust predictor of thermal sensation, underscoring the importance of focused monitoring strategies [43,44,52]. This suggests that localized monitoring could provide early signals of thermal stress before clinical symptoms manifest, enabling more precise preventive interventions.

Machine learning models enhanced this predictive capacity, allowing estimation of core body temperature and thermal comfort without the need for wearables [45,52]. Linear regression and Ridge models achieved low RMSE values (0.27 °C for rectal temperature and 0.73 °C for skin temperature), while sequential models like GRU (Gated Recurrent Unit) were most accurate under dynamic conditions. Individual characteristics such as age, weight, and height showed relevant effects on thermal prediction, and ambient temperature and humidity increased predictions of thermal stress [45,52]. These findings highlight the critical interaction between personal and environmental factors, suggesting that individualized predictive systems could be useful for anticipating thermal risks in older adults, provided they are adapted to each individual’s conditions and characteristics.

Behavioral adaptations in older adults were variable and, in some cases, insufficient to prevent prolonged exposure to extreme heat. Residents with dementia adjusted their clothing less and remained in slightly cooler environments, while those without dementia modified their clothing to maintain relative comfort [44]. Observational studies during extremely hot summers revealed that, despite increased use of air conditioning, a significant percentage of older adults remained exposed to high indoor temperatures, reflecting limitations in passive strategies and persistent risks of hyperthermia [49]. Educational interventions on hydration, breaks, protective clothing, and recognition of thermal stress symptoms significantly improved both perception and risk reduction, increasing the proportion of participants classified as “safe” and decreasing those at “very high risk” [48]. This underscores that education and awareness about thermal risk are essential but may not be sufficient on their own in vulnerable populations.

Regarding active interventions, local cooling devices and microclimate systems with ventilated vests proved effective in reducing skin temperature and thermal sensation, especially in exposed areas such as the torso, arms, and hands [50,51]. Internal reductions reached up to 3.4 °C, improving overall thermal sensation and decreasing physiological strain. Validation of these interventions through computational fluid dynamics (CFD) confirmed the utility of computational models to assess thermal mitigation strategies [54,55]. These findings support the implementation of combined interventions integrating active cooling with localized monitoring and individualized prediction.

Beyond the technical feasibility of the reviewed technologies, several practical barriers may hinder their real-world adoption. These include high acquisition and maintenance costs, limited usability for older adults with cognitive or physical impairments, and reduced accessibility in low-resource or rural settings. Long-term adherence also remains a challenge, as devices often require continuous use, charging, and digital literacy. Moreover, privacy and data protection issues associated with wearable sensors and continuous monitoring systems represent a growing concern among both users and healthcare providers. Addressing these barriers will be essential for translating technological advances into sustainable and equitable heatstroke prevention strategies.

A critical finding of this review is the absence of studies evaluating the use of wearables for heatstroke prevention in real-world settings for older adults. While these devices are commercially available and have proven effective in younger adults, no studies have shown how they could be integrated to alert older adults early about hyperthermia risks and prevent fatal outcomes [18,19,20,21,22,23,26,27]. This represents a significant research gap, given the potential of these systems to combine physiological, environmental, and behavioral data into adaptive early-warning algorithms. Some of the existing technologies, such as dual heat flux thermometers originally designed for occupational monitoring, could inform future developments aimed at older adults, provided they are adapted for usability and comfort in daily life contexts [6,36].

A significant limitation of this review is the limited evidence base, as only nine studies met the inclusion criteria. The small number of eligible studies, together with considerable heterogeneity in design, setting, participant characteristics, and outcome measures, prevented a formal meta-analysis and limits the ability to draw definitive or generalizable conclusions regarding the comparative effectiveness of interventions. Many included studies had small sample sizes, were conducted under controlled laboratory conditions, and lacked validation in real-world or long-term settings, which further reduces external validity.

In addition, most of the included literature originated from Asia and Europe, with little representation from tropical or low-resource regions. This geographic bias may influence the applicability of findings to populations exposed to different climatic, infrastructural, or socioeconomic conditions. Consequently, the results should be interpreted with caution when extrapolated to global ageing populations.

Finally, most studies focused on short-term physiological responses, such as transient changes in skin or core temperature, rather than sustained outcomes like long-term prevention of heatstroke episodes, reduction in chronic heat sensitivity, or user adherence over time. Future research should include diverse geographic and socioeconomic contexts and adopt longitudinal, ecologically valid designs to strengthen the evidence base and assess the long-term effectiveness of thermoregulation interventions in older adults.

Based on the evidence synthesized in this review, emerging technologies for thermoregulation in older adults can be categorized according to their functional level of development and application:

(a) Monitoring level: Includes wearable temperature sensors, sweat patches, infrared thermography, and environmental IoT nodes that continuously capture physiological and ambient parameters. These tools primarily enable real-time monitoring and early detection of deviations in thermal homeostasis. However, their application remains mostly limited to laboratory or controlled environments, with scarce validation in everyday contexts.
(b) Prediction level: This group encompasses machine learning models that estimate thermal sensation, core temperature, or heat strain based on physiological and environmental inputs. Examples include regression and neural network approaches (Ridge, LSTM, GRU) that outperform traditional biophysical models. These tools form the analytical bridge between raw data and individualized thermal risk prediction.
(c) Intervention level: Encompasses active cooling or heating systems, such as thermoelectric devices, ventilated clothing, and microclimatic chairs, as well as behavioral and educational strategies to enhance heat stress awareness. These technologies translate monitoring and predictive insights into actionable interventions, aiming to maintain comfort and prevent hyperthermia or hypothermia.
(d) Ecological application level: Refers to the integration of the above technologies into real-world preventive frameworks, combining wearable data, environmental monitoring, and adaptive algorithms to guide decision-making by older adults, caregivers, or healthcare systems. Currently, no study has validated such integrated systems in community or institutional settings.

Building on this classification, a coherent research roadmap can be proposed. Future investigations should progress sequentially from validating wearable monitoring systems in ecological contexts to integrating them with predictive algorithms for early detection of thermal strain, and ultimately to personalized, feedback-based interventions implemented in daily life. Large-scale longitudinal studies are needed to evaluate the long-term impact of these technologies on heat-related morbidity and mortality among older adults.

The findings suggest that combining predictive models, localized monitoring, educational strategies, and active cooling can enhance thermal safety for older adults. Future integration of wearables adapted to this population would allow personalized early alerts, with great potential to prevent adverse thermal events. Therefore, recommendations and future directions include longitudinal studies integrating physiological, behavioral, and environmental measures in older adults, evaluating the effectiveness of adaptive early-warning systems through wearables. It is also important to explore how modulatory factors such as frailty, comorbidities, polypharmacy, physical capacity, and sex affect thermal vulnerability. Finally, research should assess the implementation of these strategies in real-world settings to develop public health policies aimed at preventing heatstroke and protecting this vulnerable population. Future validation should adopt a multimodal early-warning framework combining wearable sensors, environmental IoT nodes, and machine learning algorithms to continuously estimate thermal strain in ecological contexts. This framework could enable proactive alerts for healthcare providers or caregivers, preventing heatstroke and enhancing autonomy and safety in older adults.

## 5. Conclusions

This systematic review shows that older adults exhibit a reduced physiological capacity to maintain thermal homeostasis during heat exposure, due to impaired sweating, cutaneous vasodilation, and autonomic and cardiovascular responses. These limitations promote greater heat storage and more rapid elevations in core body temperature, increasing the risk of hyperthermia, dehydration, and functional decline. At the population level, consistent increases in mortality and hospitalization among older adults are observed during episodes of high temperatures and heat waves, with heightened vulnerability at advanced ages, in women, and among individuals with comorbidities or social disadvantage.

The findings of this review reinforce the importance of implementing targeted preventive measures for older adults, including clinical surveillance strategies, community support programs, and public policies designed to reduce exposure and enhance resilience to heat. They also highlight the need for future research integrating both physiological measures and clinical outcomes, as well as evaluations of effective interventions to mitigate the impact of heat in this population in the context of climate change.

## Figures and Tables

**Table 1:** PIO Format.

Population	Older adults (≥60 years), in community, institutional, or clinical settings.
Intervention	Strategies, devices, or programs aimed at assessing or improving thermoregulation (e.g., wearables, smart clothing, community interventions during heat waves, cooling or hydration protocols).
Outcomes	Improvement in body temperature monitoring, early detection of alterations, reduction in heat- or cold-related adverse events, enhanced safety and well-being.
Research question	What interventions and technologies have been implemented in older adults to assess or improve thermoregulation, and what outcomes have they reported in terms of monitoring, prevention of adverse thermal events, and promotion of thermal well-being?

**Table 2:** Search strategy used, adapted to each of the databases.

Data Base	Search Strategies
PubMed	((“Aged” [MeSH] OR “Older adults” OR elderly OR “older people” OR seniors) AND (“Thermoregulation” [MeSH] OR thermoregulation OR “body temperature regulation” OR “heat stress disorders” [MeSH] OR “cold stress” OR “thermal stress”) AND (“Wearable Electronic Devices” [MeSH] OR wearable * OR “smart clothing” OR “sensors” OR “monitoring device *” OR “community intervention *” OR “public health intervention *”) AND (“Program Evaluation” [MeSH] OR intervention * OR technology OR prevention OR monitoring OR wellbeing OR safety))
Scopus	(TITLE-ABS-KEY(“older adults” OR elderly OR “older people” OR seniors) AND TITLE-ABS-KEY(thermoregulation OR “body temperature regulation” OR “thermal stress” OR “heat stress” OR “cold stress”) AND TITLE-ABS-KEY(wearable * OR “smart clothing” OR sensor * OR “monitoring device *” OR “community intervention *” OR “public health intervention *”) AND TITLE-ABS-KEY(intervention * OR technology OR prevention OR monitoring OR wellbeing OR safety))
ScienceDirect	(“older adults” OR elderly) AND (thermoregulation OR “thermal stress”) AND (wearable OR sensor OR “smart clothing” OR cooling)AND (“body temperature” OR “adverse events”)
WOS	TS = (“older adults” OR elderly OR “older people” OR seniors) AND TS = (thermoregulation OR “body temperature regulation” OR “thermal stress” OR “heat stress” OR “cold stress”) AND TS = (wearable * OR “smart clothing” OR sensor * OR “monitoring device *” OR “community intervention *” OR “public health intervention *”) AND TS = (intervention * OR technology OR prevention OR monitoring OR wellbeing OR safety)

**Table 3:** Characteristics of the studies included in the systematic review.

	Typology/Main Objective	Participants	Variables/Instruments	Main Findings	International Banking Institute (JBI)
[43]	Design: non-randomized experimental study. Objective: To develop predictive models of thermal sensation in older adults using field and laboratory data, and to evaluate their accuracy against existing PMV and UCB models.	N field = 1040 Age = 70–97 years Sex (f/m)%: 66.3%/33.7% N laboratory: 18 Age: 65–83 Sex (f/m): 9/9	Demographic: age, sex, self-reported health, degree of acclimatization, lifestyle habits (questionnaire) Environmental: air temperature (WSZY-1), relative humidity (10–90%), black globe temperature (TM200), air velocity (Air velocity meter 9515), illuminance (ZDS-10F-3D: Shanghai Precision Instrument Co., Ltd; Shangai, China), CO2 concentration (Testo 535), sound level (TES-1350A) Physiological: skin temperature (Pyrobutton-L) at 8 locations in the laboratory, measured every 1 min Thermal sensation: TSV using a 7-point scale (field and laboratory), subsequently recoded into 3 categories (Cool, Neutral, Warm)	In the field study, air temperature was the strongest predictor of older adults’ thermal sensation, with “Cool” at ~13 °C, “Warm” at ~30 °C, and “Neutral” around 14 °C and 28 °C. Other significant predictors included air velocity, illuminance, CO_2_ concentration, length of stay in aged-care homes, and self-reported health. The field study model achieved 56.6% overall accuracy, outperforming the PMV model (36.6%), which was biased toward “Cool.” In the lab study, five skin temperatures (head, lower arm, upper leg, chest, back) predicted thermal sensation with 76.7% accuracy, better than the UCB model (46.6%). Head and lower arm temperatures were the most important local predictors, and an air velocity threshold (~0.25 m/s) indicated adaptive behaviors that reduce heat perception. Neutral temperature ranges varied by season and health: summer 25.3–32.5 °C, mid-season 9.2–27.6 °C, winter 6.4–19.9 °C; healthy, long-stay residents tolerated cooler temperatures. A simplified head temperature-only model reached 53.4% accuracy, showing single-site measurements can be useful but less precise than multi-site models.	8/9
[44]	Design: non-randomized experimental study Objective: To evaluate the applicability of three methods for measuring thermal comfort (Predicted Mean Vote, Adaptive Comfort, and infrared thermography of extremities) in older adults living in care homes, considering differences between participants with and without dementia.	N older adults: 69 Age older adults: 60–101 years N young adults: 17 Age young adults: 18–34 years	Age, sex, clinical frailty: 7-point frailty scale (Dalhousie University, version 2007-09) Indoor environmental conditions: air temperature, relative humidity, air speed (Kestrel 3000) Clothing insulation (clo): weighted valuation of clothing ensembles Body temperature: tympanic measurement (thermo-scan, Model LF 40, Braun) Subjective thermal comfort: Thermal Sensation Vote (TSV), ASHRAE 7-point scale (−3 to +3); Thermal preference: McIntyre thermal preference scale (cooler, no change, warmer)	The study evaluated 69 older adults (34 with dementia and 35 without dementia) in 15 care homes in the UK over one year. The mean indoor temperature was 23.6 °C, with no significant differences between summer and heating periods, and clothing insulation remained stable (0.6–0.7 clo). Residents with dementia wore slightly thicker clothing (0.70 vs. 0.60 clo, p = 0.005) and were in slightly cooler spaces (23.15 °C vs. 24.07 °C, p = 0.001), while residents without dementia adjusted their clothing according to temperature. The PMV model indicated a comfort threshold between −0.51 and −0.76 PMV, with an estimated MET centered between 1.3 and 1.39, showing sensitivity to low activity levels. The adaptive approach estimated the comfort zone for non-dementia residents between 22.8 and 23.6 °C, with 80% reporting no desire to change the temperature. Infrared thermography measurements (ΔT1) between fingers and wrist correlated with comfort perception in young adults and older adults without dementia (r = 0.667–0.839, p < 0.05), but were not useful for residents with dementia; in older adults, core body temperature (+) and clothing insulation (−) were the most influential factors on ΔT1.	8/9
[45]	Design: non-randomized experimental study Objective: Develop and evaluate models for predicting body temperature (rectal and skin) in older adults (≥60 years) under different temperature and humidity conditions, and compare them with existing biological models.	N = 76 Age: ≥60 years Sex (f/m): 32/44	Rectal temperature (core): Continuous measurement using rectal thermometer (per minute) Mean skin temperature: Calculated according to ISO 2004 [46] using weighted measurements from: forehead (7%), right scapula (17.5%), upper left chest (17.5%), upper right arm (7%), right forearm (7%), left hand (5%), right anterior thigh (19%), left calf (20%) Height: Measured in centimeters (cm) Body mass: Measured in kilograms (Kg) Ambient temperature: Measured in chamber (°C) Relative humidity: Measured in chamber (%) Previous temperatures: Rectal and skin temperatures from the previous minute (input for point-wise models) Model type: Linear regression, Ridge regression, Recurrent Neural Network (RNN), Long Short-Term Memory (LSTM), Gated Recurrent Unit (GRU) Model evaluation: Root-Mean Squared Error (RMSE), Mean Bias Error (MBE), Bland–Altman Limits of Agreement, proportion of participants within clinical thresholds (0.3 °C rectal, 1.0 °C skin)	This study developed machine learning models and compared their accuracy with biophysical models to predict rectal and skin temperature in older adults. The Ridge and Linear Regression models predicted temperatures better than traditional biophysical models. Ridge Regression had the lowest root mean square error (RMSE) for rectal temperature (0.27 °C), skin (0.73 °C), and body (0.34 °C). Among sequence models, GRU was the most accurate, while RNN performed the worst. The Takahashi and Ji biophysical models predicted reasonably well; Takahashi was better for skin and body, Ji for rectal. Ridge Regression achieved 114 out of 162 participants with rectal temperature within a clinical limit of 0.3 °C and 142 out of 162 for skin within 1 °C. Most machine learning models showed mean biases (MBE) close to zero, indicating no systematic over- or underestimation. Age slightly increased the rectal temperature predicted by Ridge, while height and weight affected it variably depending on the model. High ambient temperature and humidity increased predicted temperatures, with some exceptions in GRU. Sequence models could outperform simple regression models in more dynamic conditions if trained with more data. These individualised predictions could improve heat risk monitoring and guide preventive interventions in older adults.	7/9
[47]	Design: non-randomized experimental study Objective: To explore whether facial skin temperature measured using infrared imaging can be used as an indicator of thermal sensation in older adults, and to evaluate the performance of different machine learning models in predicting that sensation.	N: 34 Age: ≥60 years, mean 83 years Sex (f/m): 20/14	Facial skin temperature (forehead, eyes, nose, cheek, chin): Testo 872 thermal imaging camera (resolution 320 × 240 px, accuracy ±2 °C, thermal sensitivity <0.05 °C). Air temperature (Ta) and relative humidity (RH): HOBO MX2302A (accuracy ±0.02 °C; ±2.5% RH; frequency: 1 min). Wind speed (v) and black globe temperature (Tg): HD32.3 (accuracy ±0.15 m/s; class 1/3 DIN; frequency: 1 min). Mean radiant temperature (Tmrt): calculated from Tg, Ta, v and physical constants. Basic information: age, gender, weight, height, clothing. Perceptions: wind chill, thermal comfort, preferences (air, humidity, solar radiation, air speed).	During the experiment, the average air temperature was 31.5 °C, relative humidity was 73.5%, wind speed was 1.5 m/s, and solar radiation was 200 W/m2, representative of summer in Guangzhou. In terms of perceived temperature and comfort ratings, the more exercise the participants did, many participants reported neutral or comfortable sensations even in warm environments, which may reflect altered thermal perception rather than true heat tolerance. In terms of environmental preferences, older adults showed low sensitivity to humidity but a marked preference for higher air velocity, indicating ventilation as key to thermal comfort. The average facial temperature was highest on the nose (35.1 °C) and lowest on the cheek (34.2 °C). The recorded range was: nose: 31.9–39.1 °C, eyes: 32.6–38.3 °C, forehead: 31.5–38.6 °C, cheek: 30.7–38.0 °C, chin: 30.8–37.8 °C. There were significant differences between facial regions (p < 0.001). The greatest effect was observed between the nose and cheeks (d = 0.813, large). Facial temperature was related to the thermal sensation: In ‘neutral’: nose 35.4 °C, eyes 35.3 °C, cheek 34.5 °C; In ‘heat’: nose 36.5 °C, eyes 36.1 °C, cheek 35.3 °C. Of the prediction models, Random Forest (RF) was the most accurate (AUC = 0.889, accuracy = 80.6%), outperforming other algorithms (SVM and LR with AUC = 0.706). For prediction with fewer measurFor prediction with fewer measurement points, the best single site was the nose (AUC = 0.825). With two points: nose + cheek (AUC = 0.856); with three points: forehead + nose + chin (AUC = 0.876); with four points: eyes + forehead + nose + chin (AUC = 0.888). The nose was present in all optimal combinations, making it the key site for monitoring thermal sensation in older adults.	9/9
[48]	Design: non-randomized experimental study Objective: Evaluate the effectiveness of a sustainable heat stress prevention program (Sustainable Prevention Programme) in reducing excessive heat among agricultural workers over the age of 60.	N = 120 Age: ≥60 years Sex (f/m): 41/79	Perceived heat strain: Observational-Perceptual Heat Strain Risk Assessment (OPHSRA) Individual heat strain: Heat Strain Score Index (HSSI) Knowledge and behaviors regarding heat prevention: Socio-demographic questionnaire and interviews Environmental exposure: Measurement of temperature, humidity, and solar exposure	The intervention consisted of education on hydration, breaks, protective clothing, and recognition of heat stress symptoms, delivered over six weeks in interactive sessions. The results showed significant improvements in the intervention group: the percentage of participants classified as “safe” on the HSSI increased from 26.7% to 45.0% (p = 0.007), while those considered “at risk” decreased from 25.0% to 15.0% (p = 0.014). Similarly, the OPHSRA indicated an increase in low risk from 18.3% to 31.7% (p = 0.003) and a decrease in very high risk from 11.7% to 5.0% (p = 0.001). The control group showed no significant changes. In the OPHSRA, the ‘low’ risk increased from 18.3% to 31.7% after the program (p = 0.003). The ‘very high’ risk in OPHSRA was reduced from 11.7% to 5.0% in the intervention group (p = 0.001). The intervention tripled the odds of lower heat stress incidence (OR = 3.38; p < 0.001). Being male was associated with a higher risk of heat stress (OR = 1.55; p = 0.013). Each additional hour of exposure to high temperatures increased the risk by 9% (OR = 1.09; p = 0.003). Having hypertension increased the risk of heat stress by 68% (OR = 1.68; p = 0.006), and diabetes by 57% (OR = 1.57; p = 0.026). Age and work experience were associated with a reduced risk of heat stress (OR = 0.95 and 0.97; p = 0.039 and 0.016, respectively).	9/9
[49]	Design: Observational Objective: Investigate differences in air conditioning use and indoor thermal demand among older adults during an extremely hot summer (2022) and a normal summer (2023) in Chongqing, China.	N = 26 Age: 84 ± 7 years Sex (f/m): 12/14	Age, sex, height, weight, BMI: Initial questionnaire/anthropometric data. Indoor air temperature: Hobo temperature/relative humidity data logger—UX100-003. Indoor relative humidity: Hobo temperature/relative humidity data logger—UX100-003. Outdoor air temperature: Hobo temperature/relative humidity data logger—UX100-003 (installed in open staircase). Air conditioner (RAC) operation time: Cloud platform connected via IoT to RAC. Air conditioner (RAC) activation frequency: Cloud platform connected via IoT to RAC. Air conditioner (RAC) setpoint temperature: Cloud platform connected via IoT to RAC.	During the extremely hot summer, the outdoor temperature exceeded 35 °C for 19 days, and the average indoor temperature was higher (29.8 °C vs. 28.4 °C in a normal summer). Thermal comfort was limited: only 12% of the time was within the comfortable range in extreme summer with air conditioning on, compared to 34% in a normal summer. Air conditioning (RAC) use was 2.4 times higher in extreme summer (26% of the time vs. 11% in normal summer), with more prolonged episodes (>10 h). The elderly turned on the RAC more often in extreme summer (44% multiple times/day), but one-third never used it, even in intense heat. The RAC was turned on at higher indoor temperatures in extreme summer (29.8 °C vs. 28.3 °C, p < 0.001). In normal summer, frequent switching on before dawn suggests possible sleep interruptions. Set points were higher in extreme summer (28.8 °C, preference 30 °C) than in normal summer (26.7 °C, preference 26 °C). Predictive analysis showed that each 1 °C increase in outdoor temperature increased the probability of RAC use by 6.9% (extreme summer) and 8.4% (normal summer). However, even at >40 °C, maximum use reached only 60%.	10/11
[50]	Design: non-randomized experimental study Objective: To evaluate how the use of three local cooling devices (table fan, air jacket, and evaporative device) affects skin temperature and thermal sensation in older adults, and to explore the relationship between skin temperature and thermal sensation.	N = 26 Age: ~70.8 years Sex (f/m): 19/7	Local skin temperature: iButton sensors (DS1923, accuracy ± 0.5 °C) Core temperature (tympanic): Ear thermometer (Braun IRT6520, accuracy ± 0.2 °C) Overall thermal sensation: Questionnaire, 7-point scale (−3 cold to +3 hot) Local thermal sensation (head, torso, limbs, extremities): Questionnaire, 7-point scale (−3 cold to +3 hot) Air temperature (Ta): TinyTag 2 Plus data loggers (accuracy ±0.5 °C) Relative humidity (RH): TinyTag 2 Plus data loggers (accuracy ± 3%) Operative temperature (To): ComfortSense probes (accuracy ± 0.2 °C) Air velocity: ComfortSense probes (accuracy ± 0.02 m/s) Cooling effect on body segments (heat loss): Thermal manikin with 27 body segments, electricity consumption measurements Power consumption of cooling devices: Device specifications + manikin test measurements	Ambient temperature (Ta) significantly influenced skin temperature (p < 0.05), whereas relative humidity did not (p > 0.05). In older adults, distal regions (head, hands, feet) showed lower skin temperatures than in younger adults. The MST (mean skin temperature) decreased significantly in the first 10 min after using cooling devices, with reductions <0.5 °C (p < 0.05). The core temperature decreased significantly after using devices compared to the uniform condition (p < 0.05), remaining stable during the cooling phase. Significant local temperature decreases: Fan: chest, forearm and palm (up to −0.8 °C and −0.6 °C in slightly warm conditions, p < 0.01). Evaporative device: forearm (−0.9 °C) and chest (−1.1 °C) in warm conditions (p < 0.01). Air jacket: chest and lumbar region (−0.6 to −0.7 °C, p < 0.01). The evaporative and air jacket devices were more effective in warm environments, while the fan was more effective in slightly warm environments. Cooling mainly affected exposed parts (chest, arms, hands, upper and lower back), while unexposed parts (feet, calves) remained further from the neutral range. The thermal sensation (TSV) was significantly reduced in exposed areas (p < 0.05), with greatest influence on overall perception. The strongest correlation between skin temperature and thermal sensation was observed in the extremities and hands, especially in the fingers (highest r). The thermosensory mean skin temperature (TMST) was a better predictor of overall thermal sensation than the classic MST (p < 0.01).	8/9
[51]	Design: non-randomized experimental study Objective: To evaluate the effect of a personal comfort system (PCS) based on a microclimatic chair combined with a ventilated vest on the thermal sensation (TS) of elderly people in hot conditions, optimizing thermal comfort and reducing energy consumption.	N = 29 Age: 70.9 ± 5.8 years Sex: not specified	Whole-body Thermal Sensation, TS: Subjective voting on ASHRAE 7-point scale Mean and segmental skin temperature: Temperature sensors in thermal manikin and experimental human data Air velocity and temperature in microclimate: Omnidirectional anemometers and thermocouples Ventilation and air flow in vest: Fan flow rate and flow sensors Energy consumption: Electrical power calculations for compressors and fans	The temperature predicted by Computational Fluid Dynamics (CFD) and the vest model matched experimental measurements within a range of ±0.5 °C. The predicted air velocity matched measurements within ±0.2 m/s. Activation of the vest reduced internal temperatures in the torso by up to 3.4 °C (Personalized Conditioning System (PCS) switched on). The maximum difference between the predicted and measured temperature in the vest was 0.6 °C. Impact on skin temperature and thermal sensation in older adults: With PCS alone (33 °C, 22 °C, 17–21 l/s): mean skin temperature (Tsk) 34.0–34.1 °C, TS ≈ 0.8–0.9 (slightly warm). PCS + vest (33 °C, 22 °C, PCS 17–21 l/s, vest 6–11 l/s): Tsk 33.7–34.0 °C, TS 0.5–0.8 (neutral to slightly warm). PCS + vest reduces trunk Tsk by up to 1.7 °C compared to PCS alone. At 29 °C, PCS alone (22 °C, 17–21 l/s): Tsk 33.3–33.4 °C, TS ≈ 0 to −0.1 (neutral). PCS + vest (29 °C, 22 °C, PCS 21 l/s, vest 11 l/s): Tsk 32.8 °C, TS ≈ −0.4 (slightly cool). PCS creates a cool microclimate around the body (~28–30 °C). Vest extracts cool air from the microclimate, which can raise the temperature in the lower body by up to 0.5 °C, but does not significantly affect the inner skin. Wider microclimate with high-flow PCS and low-flow vest; finer microclimate with activated vest and lower flow.	8/9
[52]	Design: non-randomized experimental study Objective: Develop and validate a simplified machine learning model to predict individual thermal comfort in older adults without using wearable devices, using personal, environmental, and temporal variables.	N = 8 Age: ≥60 years Sex: Not specified	Physical activity/metabolic rate (MET): machine learning model using proxy variables Individual thermal comfort (PMV): Calculated with PMV considering air temperature, relative humidity (SHT30 sensors), assumed mean radiant temperature, air velocity (0.1 m/s), clothing insulation (0.5–1 clo), and metabolic rate Height, Weight, BMI, Percent body fat (PBF), Skeletal muscle mass (SMM), Body fat mass (BFM), Visceral fat level (VFL): Inbody dial WH20 N body composition analyzer Air temperature, Relative humidity: IoT sensor SHT30	Welch’s ANOVA showed significant differences in thermal comfort among the eight older adults even under similar thermal environments (Levene p < 0.001; ANOVA p < 0.05 in all seasons). The Random Forest model accurately predicts metabolic activity and thermal comfort without the need for portable devices. Mean absolute error (MAE) of metabolic activity (MET): 0.097–0.106 depending on the season, average 0.098. MAE of thermal comfort (PMV): 0.039–0.058 depending on the season, average 0.048. The prediction tends to overestimate when actual values are low and underestimate when they are high. It is recommended to adjust predictions according to the population mean to improve accuracy.	9/9

Note: The thermometric instruments and indices reported in the included studies were standardized according to ISO 9886:2004 and their original methodological publications (see References [36,37,38,39,40,41,42]).

**Table 4:** Quality assessment of quasi-experimental studies (JBI checklist).

	Q1	Q2	Q3	Q4	Q5	Q6	Q7	Q8	Q9
[43]	+	+	+	-	+	+	+	+	+
[44]	+	+	+	-	+	+	+	+	+
[45]	+	+	-	+	-	+	+	+	+
[47]	+	+	+	+	+	+	+	+	+
[48]	+	+	+	+	+	+	+	+	+
[50]	+	+	+	-	+	+	+	+	+
[51]	+	+	-	+	+	+	+	+	+
[52]	+	+	+	+	+	+	+	+	+

**Table 5:** Quality assessment of the observational study (JBI checklist).

	Q1	Q2	Q3	Q4	Q5	Q6	Q7	Q8	Q9	Q10	Q11
[49]	+	+	+	+	+	+	+	+	+	-	+

## Data Availability

No new data were created or analyzed in this study.

## Supplementary Materials

The following supporting information can be downloaded at: https://www.mdpi.com/article/10.3390/s25227058/s1, Table S1: PRISMA 2020 Checklist [56].
